# Genomics, Proteomics, and Antifungal Activity of Chitinase from the Antarctic Marine Bacterium *Curtobacterium* sp. CBMAI 2942

**DOI:** 10.3390/ijms25179250

**Published:** 2024-08-26

**Authors:** Yesenia Melissa Santa-Cruz Vasquez, Luis Gabriel Cueva-Yesquen, Alysson Wagner Fernandes Duarte, Luiz Henrique Rosa, Rodrigo Valladão, Adriana Rios Lopes, Rafaella Costa Bonugli-Santos, Valéria Maia de Oliveira

**Affiliations:** 1 Divisão de Recursos Microbianos, Centro Pluridisciplinar de Pesquisas Químicas, Biológicas e Agrícolas (CPQBA), Universidade Estadual de Campinas (UNICAMP), Paulínia 13148-218, SP, Brazil; melly89sc@gmail.com (Y.M.S.-C.V.); luisg_cueva@yahoo.es (L.G.C.-Y.); 2 Institute of Biology, Campinas State University (UNICAMP), Campinas 13083-970, SP, Brazil; 3 Complexo de Ciências Médicas e de Enfermagem, Universidade Federal de Alagoas, Campus Arapiraca, Arapiraca 57309-005, AL, Brazil; 4 Instituto de Ciências Biológicas, Universidade Federal de Minas Gerais, Belo Horizonte 31270-901, MG, Brazil; lhrosa@icb.ufmg.br; 5 Laboratory of Biochemistry, Instituto Butantan, São Paulo 05585-000, SP, Brazil; rodrigovalladao3@gmail.com (R.V.); adriana.lopes@butantan.gov.br (A.R.L.); 6 Instituto Latino Americano de Ciências da Vida e da Natureza (ILACVN), Universidade Federal da Integração Latino-Americana (UNILA), Foz do Iguaçu 85870-650, PR, Brazil; rafaella.santos@unila.edu.br

**Keywords:** antifungal activity, Antarctic environment, marine bacteria, bacterial chitinases, *Curtobacterium*, optimization of enzyme production

## Abstract

This study aimed to evaluate the genomic profile of the Antarctic marine *Curtobacterium* sp. CBMAI 2942, as well as to optimize the conditions for chitinase production and antifungal potential for biological control. Assembly and annotation of the genome confirmed the genomic potential for chitinase synthesis, revealing two ChBDs of chitin binding (Chi C). The optimization enzyme production using an experimental design resulted in a 3.7-fold increase in chitinase production. The chitinase enzyme was identified by SDS-PAGE and confirmed through mass spectrometry analysis. The enzymatic extract obtained using acetone showed antifungal activity against the phytopathogenic fungus *Aspergillus* sp. series *Nigri* CBMAI 1846. The genetic capability of *Curtobacterium* sp. CBMAI 2942 for chitin degradation was confirmed through genomic analysis. The basal culture medium was adjusted, and the chitinase produced by this isolate from Antarctica showed significant inhibition against *Aspergillus* sp. *Nigri* series CBMAI 1846, which is a tomato phytopathogenic fungus. This suggests that this marine bacterium could potentially be used as a biological control of agricultural pests.

## 1. Introduction

Chitinases (EC 3.2.1.14) are enzymes that act in the hydrolysis of β-type 1–4 bonds of N-acetyl-D-glucosamine (GlcNAc) of chitin, the second most available carbohydrate in nature, being fundamental for its degradation and nutrient cycling [1]. Based on amino acid sequence similarity, chitinases are grouped into three families: GH18, GH19, and GH20 of glycosyl hydrolases [2,3]. Different biotechnological applications of chitinases are reported, such as the control of agricultural pests [4], production of derivatives such as N-acetyl-D-glucosamine from shrimps [5], of chitosan and chitooligosaccharides [6,7,8], and of ethanol [9].

A differential of the chitinases produced by Antarctic bacteria is that they are enzymes with optimal activity in processes that occur at low temperatures and show significant advantages in industrial applications, such as the treatment of waste with chitin, as well as the biological control of phytopathogens [10] in cold weather environments and the control of microbiological contamination in refrigerated foods, increasing their shelf life [11]. Much research has focused on the study of chitinase production by microorganisms from thermophilic environments [12,13,14]; however, little interest has been directed towards the search for chitinase-producing microorganisms adapted to cold environments such as Antarctica.

In this context, cold-adapted enzymes have high catalytic efficiency in processes that occur at low and moderate temperatures. The use of psychrophilic enzymes in industrial processes that occur at low temperatures offers some advantages, such as a reduction in substrate uptake in specific enzymatic processes, since, in general, they have a higher substrate specificity compared to enzymes from mesophilic microorganisms [15]. This advantage is quite interesting in industrial processes due to the reduction of production costs [15].

A range of different studies have been carried out in the search for the biotechnological potential of Antarctic microorganisms, and many of them have successfully shown the production of psychrophilic and psychrotolerant enzymes, such as lipase, amylase, and protease [16], and chitinase [17], besides other applications such as phosphate solubilization for agricultural use [18]. These have employed a myriad of methodological approaches to gain deep knowledge of the physiology of production, genetics, and potential applications of these molecules. Nonetheless, studies reporting chitinases from Antarctic microorganisms are scarce. The present study aimed to genetically identify and chemically characterize the genes and enzymes responsible for chitinase production in the Antarctic strain *Curtobacterium* sp. CBMAI 2942, isolated from bryozoans at Punta Hannah–Livingston Island, as well as to evaluate its antifungal activity for biological control purposes.

## 2. Results

### 2.1. Sequencing, Assembly, and Taxonomic Affiliation of Marine Curtobacterium sp. CBMAI 2942 Genome

The de novo assembly allowed the reconstruction of the *Curtobacterium* sp. CBMAI 2942 genome in a single circular sequence. The quality of the assembly was assessed by calculating the number of contigs, the N50 value, completeness, and degree of contamination (Table 1). Metrics considered “Gold standards”, such as the ANI (Average Nucleotide Identity) and digital DNA–DNA hybridization (dDDH), often used to define the boundaries of bacterial species, were determined (Table 2). The calculated values of ANI (87.70) and dDDH (33.60) between *Curtobacterium* sp. CBMAI 2942 and the related reference genomes suggest that it is a potential new species of the *Curtobacterium* genus.

**Figure 1:**
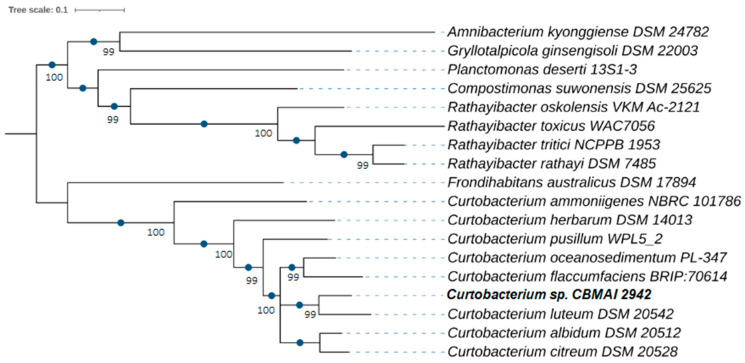
Genome-based phylogenetic tree constructed with genomic sequences of marine *Curtobacterium*, including the bacterium *Curtobacterium* sp. CBMAI 2942. Evolutionary distances were calculated from 400 ubiquitous and phylogenetically informative proteins. Orthologs of these proteins were detected using Diamond. Several sequence alignments of these proteins were generated using MAFFT. The final construction of the tree was performed using FastTree. The numbers on the branches are genetic distances.

**Figure 2:**
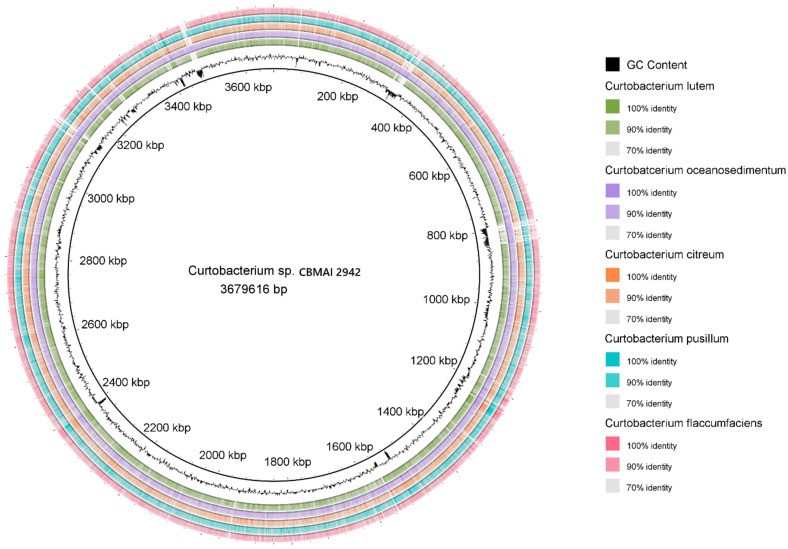
Circular representation of sequences of the complete genome of marine *Curtobacterium* sp. CBMAI 2942 and closely related species. The inner ring represents the genome of the bacterial strain CBMAI 2942 with the corresponding genetic coordinates. The colored rings (from the inner to the outer ring) represent GC content and complete genomic sequences of species *C. luteum*, *C. oceanosedimentum*, *C*. *citreum*, *C*. *pusillum*, and *C*. *flaccumfaciens.*

The evolutionary relationships were reconstructed by a phylogenomic analysis (Figure 1), using 400 orthologous genes. The tree topology confirmed the taxonomic relatedness observed by the ANI and dDDH calculations, indicating that *Curtobacterium luteum* is the most closely related species to *Curtobacterium* sp. CBMAI 2942. Another comparative analysis at the genomic level was conducted with BRIG (BLAST Ring Image Generator) software (https://sourceforge.net/projects/brig/ accessed 4 March 2022), which allowed us to determine and visualize variable and conserved genomic regions between strain CBMAI 2942 and the phylogenetically closest species (Figure 2).

### 2.2. Genomic Potential for Chitin Degradation

Functional annotation using KEGG and EggNOG databases allowed us to identify genes involved in chitin degradation in the genome of *Curtobacterium* sp. CBMAI 2942. Two sequences of approximately 1310 bp and 1553 bp associated with the chitin-binding domain (ChBD) of chitinase C (Chi C) (COG3979) were found (Table 3; Figure S1). Partial retrieval of encoding sequences may be associated with limitations derived from the high-throughput sequencing, algorithms used in genome assembly, and database robustness.

### 2.3. Production of Chitinase in Liquid Medium

**Figure 3:**
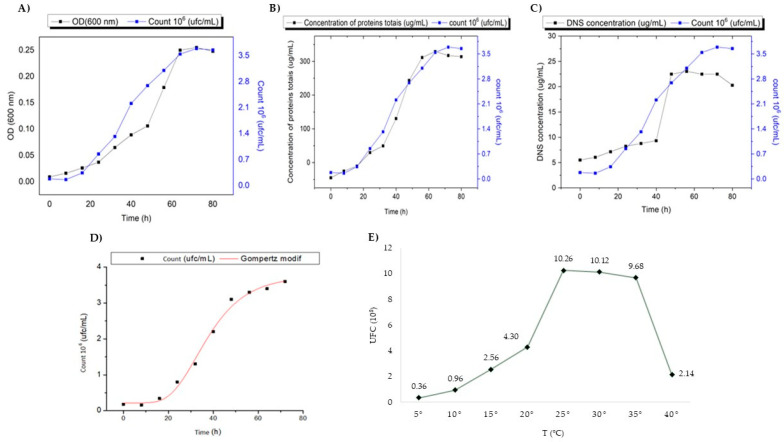
Growth curve of *Curtobacterium* sp. CBMAI 2942 in liquid culture medium with colloidal chitin (2%), incubated at 15 °C, 150 rpm for 80 h. (**A**) Biomass; (**B**) total proteins; (**C**) chitinase activity; (**D**) Gompertz model; (**E**) influence of temperature (5 °C to 40 °C) on growth for 72 h of incubation and 150 rpm. According to the mathematical model of Gompertz, *Curtobacterium* sp. CBMAI 2942 showed a rapid growth rate of 0.11 (CFU/m/t), 18.68 h of lag time, and a generation time of 6.37 h at 15 °C and 150 rpm in culture medium with colloidal chitin (2%). The Antarctic bacterial strain *Curtobacterium* sp. CBMAI 2942 showed a typical growth of psychrotolerant microorganisms, which have optimum growth temperatures up to 25–35 °C (there were no statistically significant differences [*p* > 0.05] according to Tukey test subgroups: a1: 5 and 10 °C; a2: 40 and 15 °C; a3: 20 °C; a4: 25, 30, and 35 °C) (Figure 3E).

The maximum biomass of the *Curtobacterium* sp. CBMAI 2942 was observed at 80 h of incubation (Figure 3A), and the highest value of total proteins was 329.01 µg/mL in the stationary phase (Figure 3B). Quantification of chitinase by reducing sugars (N-acetylglucosamine) showed an optimal production at 80 h of incubation with 26.46 U/L (Figure 3C). The modified Gompertz bacterial model used in the present study showed a significant correlation coefficient (R^2^ = 0.99) (Figure 3D).

### 2.4. Optimization of Chitinase Production

The statistical optimization design led to the improvement of chitinase production, resulting in a 3.7-fold increase compared to the chitinase production of the experiment without enhancement (Table 4). The strategy began with two Plackett–Burman (PB) designs to select variables for cultivation and adjust concentration ranges, followed by a Central Composite Design (CCD) aimed at optimization. To assess the effect of cultivation time on production, the tests were conducted with 96 h and 120 h of incubation. The first PB experiment showed a statistically significant effect of the variables colloidal chitin, peptone, KH_2_PO_4_, and MgSO_4_·7H_2_O, with a positive effect (*p* ˂ 0.1) in the production of chitinase by *Curtobacterium* sp. CBMAI 2942 at 96 h of incubation. Only two variables showed statistically significant effects at 96 to 120 h of incubation, colloidal chitin and peptone, both with positive effects (*p* ˂ 0.1) (Tables S1 and S2). Variables with a negative effect were excluded from cultivation, and to determine the optimal concentration of significant variables, concentrations were increased in the second PB design. In both experiments, the pH showed a negative effect, so it was fixed at the lowest value evaluated (6.0) for subsequent experiments.

The second PB design indicated that increasing peptone did not have a positive effect on enzyme activity. However, colloidal chitin showed a positive effect and was identified as the main independent variable influencing enzyme production (Tables S3–S5).

Following the evaluation of the cultivation conditions, a CCD2^3^ was performed. This experimental design included a wider range of colloidal chitin (0.31–6.19 g/L), as well as the variables peptone (0.06–0.74 g/L) and KH_2_PO_4_ (0.1–0.6 g/L); the independent variables temperature, pH, and MgSO_4_·7H_2_O were then established at 25 °C, 6.0, and 0.5 g/L, respectively. This new trial resulted in higher activity compared to previous productions, enabling an increase in chitinase production to 99.19 U/L (Table 5).

The CCD 2^3^ was carried out at two incubation times, 96 h and 120 h, and both showed interesting results (Table 5), with experimental values close to the predicted values (Figure S2). At both time points, peptone had a significantly negative effect, with higher concentrations resulting in lower production. In contrast, colloidal chitin had a significant positive impact on enzyme production (Tables S6 and S7). The mathematical model was used to evaluate process optimization over 120 h, as the best activities were achieved under these conditions.

**Figure 4:**
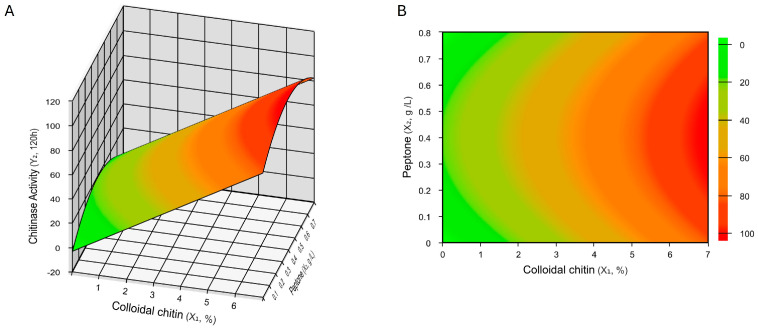
Contour response (**A**) and surface response (**B**) plots (CCD2^3^) for chitinase production by *Curtobacterium* sp. CBMAI 2942 after 120 h of incubation (*p* < 0.05). The plots illustrate the influences of two factors (colloidal chitin and peptone) with constant value of the third factor (0.35 g/L KH_2_PO_4_) on chitinase production.

The evaluation of chitinase production response at 120 h through analysis of variance (ANOVA) demonstrated that the mathematical model is significant by Fisher’s test, with an experimental value superior to the tabulated F: F 2; 14; 0.05 = 19.42 (Table S8). The regression coefficient (R^2^) was 94% (Table S8), suggesting model adequacy and showing that the model is workable and can be accepted. The equation of the parametrized model obtained is as follows: chitinase activity at 120 h = 58.43 + 21.28 (colloidal chitin) − 5.55 (peptone)^2^ (Table S7). The mathematical model was used to design the response and contour surfaces for chitinase production by *Curtobacterium* sp. CBMAI 2942 at 120 h (Figure 4). According to Figure 4, the experimental coverage was insufficient to encompass the entire model surface. This resulted in a partial view of the response, where only a gradient was observed rather than a complete view of the surface. Analysis of desirability functions [19] revealed that the optimal values were colloidal chitin 6.19%, peptone 0.40 g, and KH_2_PO_4_ 0.35 g (Figure S3). These values correspond exactly to assay 10, which produced the best response, indicating successful optimization of the culture medium.

**Figure 5:**
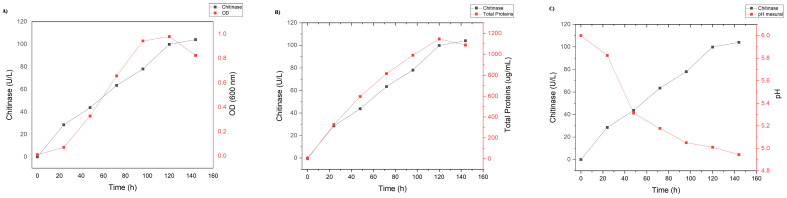
Experimental validation of chitinase production by *Curtobacterium* sp. CBMAI 2942 from time 0 to 144 h. (**A**) Chitinase production vs. OD; (**B**) chitinase production vs. total proteins (**C**); chitinase production vs. pH.

At the end of the improvement process, a 3.5-fold increase (86.27 U/L) at 96 h and a 3.7-fold increase (99.19 U/L) at 120 h of incubation in chitinase production were obtained compared to that obtained without experimental design (26.46 U/L). Three verification experiments were performed using the condition that achieved the highest CCD activity (assay 10), with an activity greater than 100 U/L (Table 5). Furthermore, the experimental validation showed that as chitinase production increases, cell growth and total protein concentration also increase (Figure 5).

### 2.5. Isolation and Proteomic Analysis of Chitinase

**Figure 6:**
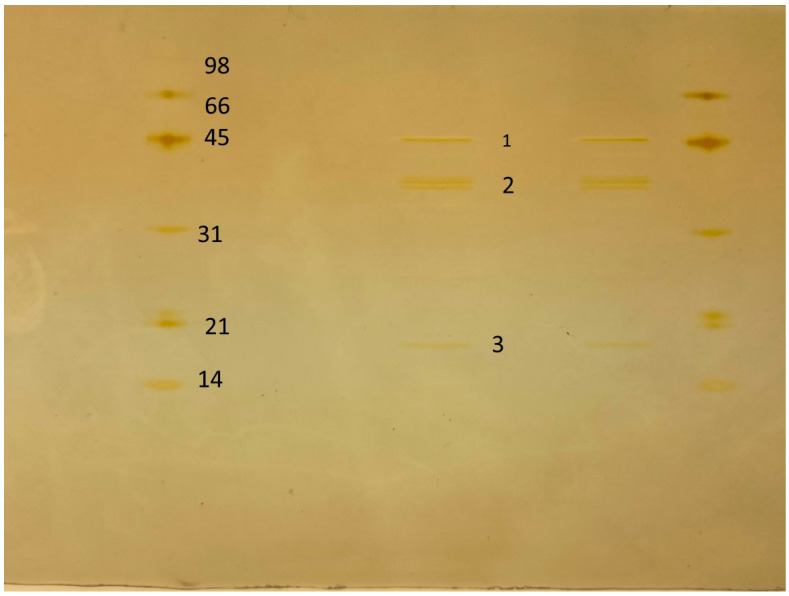
SDS-PAGE of the chitinase sample on a silver-stained 12% polyacrylamide gel evidencing three main protein bands marked (1, 2, and 3), which were analyzed by mass spectrometry.

Chitinase sample was enriched in three main protein bands: 54.1 kDa, 41.2 kDa, and 16.7 kDa (Figure 6). The three bands were isolated and submitted to proteomic analyses. Colloidal chitin and 4-Methylumbelliferyl β-D-N,N′,N″-triacetylchitotrioside were hydrolyzed by the enzyme (6.8 mU/mL using 4-Methylumbelliferyl β-D-N,N′,N″-triacetylchitotrioside as substrates). All protein bands were identified as chitinase fragments, indicating the expression of distinct chitinases and the isolation of this activity.

### 2.6. Antifungal Activity of Chitinase Produced by Curtobacterium sp. CBMAI 2942

The enzyme extract of the growth culture of *Curtobacterium* sp. CBMAI 2942, precipitated with acetone, showed antifungal activity against *Aspergillus* sp. series *Nigri* CBMAI 1846 (tomato isolate, Figure S4), with an inhibition halo of 26 mm (Figure S4A). However, it did not show activity against *Botrytis cinerea* CBMAI 0863 (grape isolate) (Figure S4B), *Fusarium complex incarnatum-equisetii* CBMAI 1981 (mango pathogen) (Figure S4C), or *Fusarium complex oxysporum* CBMAI 1274 (soil isolate) (Figure S4D). Microscopic analysis of fungal mycelium treated and not treated with the enzyme extract confirmed that the chitinase produced by *Curtobacterium* sp. CBMAI 2942 can degrade the chitin that forms the fungal cell wall, an important structure at the time of infection (Figure S5).

## 3. Discussion

The genus *Curtobacterium* has been isolated from different environments such as soil, water, and plants, among others [20,21]. Some *Curtobacterium* species have been related to the inhibition [22] or promotion of phytopathogenic fungi [23]. The latest studies have focused on the pathogenicity potential in crops [24] and on demonstrating its endophytic and epiphytic capacity [25,26,27]. The ecological versatility of *Curtobacterium* can be explained by genomic traits associated with the metabolisms of a wide variety of carbohydrates, including complex oligosaccharides [28]. This genus is classified within the phylum Actinomycetota and the family Microbacteriaceae, and it is considered mainly as a cosmopolitan that contributes to the decomposition of organic matter [29].

In this study, the strain *Curtobacterium* sp. CBMAI 2942, isolated from a bryozoan sample collected in Maritime Antarctica, shared 87.70% of ANI and 33.60% of dDDH with the closest species *C. luteum* DSM 20542^T^, which suggests that it would be a potential new species. However, it is necessary to perform a complete chemotaxonomic and biochemical characterization to describe this bacterium as a new species of the genus *Curtobacterium*. A recent genomic study of the genus *Curtobacterium* proposed 51 potential genomospecies from ANI calculations of genomes available on the NCBI database [24]. The production of chitinase by *Curtobacterium* sp. CBMAI 2942 was observed in a previous study of our research group [17], which might be related to the adaptation of the bacterium towards using the waste generated by Antarctic krill [30,31] or Bryozoa, specifically due to the protective structure stoblasts [32], as a carbon source. In addition, a study conducted by Dimkic (2021) [33] also showed chitinolytic activity of a *Curtobacterium* strain isolated from soybean leaves grown in Brazil. This latter study found the constitutive occurrence of glycosyl hydrolases (GHs) by determining the core genome together with 50 other *Curtobacterium* genomes, which is evidence of the potential for degradation of diverse polysaccharides.

The functional annotation of the *Curtobacterium* sp. CBMAI 2942 genome revealed the presence of genes involved in the degradation of chitin and named two chitin-binding domains (ChtBDs), classified as chitinase A and considered a multidomain protein. These findings confirmed the genetic potential for chitinase production by *Curtobacterium* sp. CBMAI 2942 as observed previously in biochemical assays. This same result was described in *Pseudoalteromonas* sp. DL-6, a psychrophilic bacterium isolated from marine sediments in the Bohai Sea, where two ChtBDs were annotated [34]. The chitinase genes have also been reported in plants grown in cold environments [35,36], confirming the adaptation potential of these enzymes at low temperatures. The presence of these domains was related to the increased affinity and degradation efficiency of chitin [37], which suggest the biotechnological potential of the strain *Curtobacterium* sp. CBMAI 2942 to produce chitinase at low temperatures.

In a previous study, Lonhienne and collaborators [38] reported that the strain *Arthrobacter* sp. TAD20, a bacterium isolated from marine sediments from the Dumont d’Urville Antarctic station, presents two types of chitinase genes, A and B, with two and one chitin-binding domains, respectively. In another study carried out by Orikoshi et al. [39], the authors found three types of chitinase genes in the genome of the bacterium *Alteromonas* sp. strain O-7, and in all of them, the ChtBD type 3 domain was observed, suggesting that this type of domain is essential for the efficient hydrolysis of the insoluble chitin and greater catalytic activity at low temperatures. Genetic potential for the synthesis of an enzyme with high biotechnological value, even at low temperatures, can be optimized through the heterologous expression [11,40] and, thus, boosting its implementation at industrial scales.

The maximum production of chitinase by *Curtobacterium* sp. CBMAI 2942 was 26.46 U/L in 80 h at low temperature (15 °C). This yield was considered high when compared with the bacterium *Pseudomonas* sp. GWSMS-1, which yielded 15.00 U/L, during 6 days at 20 °C [10]. In the industry, the fermentation time is an important and differential aspect in the production of enzymes, and in this study, the time required for maximum chitinase production was approximately 4 days, compared to mesophilic microorganisms, which usually require 6 to 7 days for optimal production, as is the case with *Streptomyces griseorubens* C9 [13] and *Bacillus pumilus* [41]. Other studies have reported results similar to ours, such as the one conducted with the bacterial strain *Cohnella* sp. A01, which showed an excellent production of a thermostable chitinase after 72 h [12].

Microbial growth modeling is used for prediction methods, especially in the field of fermentation and biotechnological processes. One of the most widely used models in predictive microbiology is Gompertz [42,43]. In the study carried out by Harish et al. [44], the authors analyzed three growth models (Logistic, Richards, and Gompertz) using the bacterium *Oceanimonas* sp. BPMS22, demonstrating that the best microbial growth model was modified by Gompertz. In the present study, this same model was used, and an R2 value of 0.99 was obtained, demonstrating an optimal fit. The Gompertz model [41] determines the microbial growth times in four phases (lag, exponential–log, stationary, and death); each phase varies in time length for each microorganism. One of the most important phases in this model is the lag phase (lag phase) in which the microbial metabolism is prepared to generate all the necessary components for cell growth [45].

For the study of microbial growth, temperature is fundamental; it is a widely monitored parameter in the industrial production of microbial enzymes [46]. In the case of psychrophilic bacteria, they usually show an optimum growth temperature lower than 20 °C [47]. The strain *Curtobacterium* sp. CBMAI 2942, although isolated from the Antarctic environment, demonstrated a growth temperature range of 5–40 °C, with an optimum temperature of 25–35 °C, characteristic of a psychrotolerant mesophile [48]. This aspect is of great relevance from the biotechnological point of view. A different result was recorded by Kuddus and Ramteke [49], with the psychrophilic strain *Curtobacterium luteum*, isolated from soils in the western Himalayas, with the best microbial growth temperature at 15 °C and a reduction in biomass when cultivated above 20 °C. However, these authors evaluated the production of metalloproteases and not chitinases. To achieve a high enzymatic production, it is important to define the incubation time, since a short period does not define the maximum production, and a long incubation time can lead to a decrease in enzyme production. In the present study, the maximum production of chitinase was coincident with the stationary phase based on cell counting. This result showed a correlation between the increased growth and enhanced production of chitinase by *Curtobacterium* sp. CBMAI 2942.

The experimental design was used for the optimization of chitinase production. The strategy began with factor screening using PB. This methodology ensures that all possible influences are considered, and the most significant variables are identified. Neglecting this initial screening step can lead to missing important variables, resulting in suboptimal optimization or failure to achieve the desired results. Production was then assessed using a CCD, an experimental design methodology that allows for a detailed evaluation of interactions among a reduced number of variables selected from previous steps. This approach can result in a mathematical model that the authors can use for optimization processes [5,50]. The strategy employed was highly effective for the optimization of chitinase production and resulted in a mathematical model that can be used for future optimizations. The results showed that colloidal chitin is an excellent carbon source to induce chitinase production. Similar results were observed for *Streptomyces griseorubens* C9 [13], *Cohnella* sp. A01 [12], *Gossypium* sp. (Cotton) [51], *Pseudomonas* sp. GWSMS-1 [10], and *Arthrobacter psychrochitiniphilus* 492 [17]. Also, peptone, added as a source of organic nitrogen, significantly increased chitinase production by *Curtobacterium* sp. CBMAI 2942; this may be related to protein synthesis and cell mass generation [52]. Among other macronutrients that had a significant positive effect on chitinase production by *Curtobacterium* sp. CBMAI 2942, KH_2_PO_4_ was shown to be the best source of phosphorus, used in the metabolism of synthesis of nucleic acids, phospholipids, and coenzymes [53]. Similar results were found for the bacteria *Bacillus cereus* GA6 [49], *Cohnella* sp. A01 [12], and *Streptomyces griseorubens* C9 [13]. MgSO_4_·7H_2_O had a significant positive effect as a source of sulfur, necessary to maintain the structure of amino acids used in protein synthesis; the same sulfur source was important in the production of chitinase by *Bacillus licheniformis* AT6 [54].

Temperature and pH are relevant factors in microbial growth since each microorganism is adapted to specific environmental conditions [52]. In this study, the optimal temperature for bacterial growth and chitinase production ranged from 25 to 35 °C (Figure 3E). Similar findings were reported for other cold-adapted bacteria, such as *Sanguibacter antarcticus* KOPRI 21702, isolated from King George Island in Antarctica [55], and *Pedobacter* sp. PR-M6, a psychotolerant bacterium [56]. Besides the increased production of chitinase, those ideal conditions favored the secretion of chitinases which were active on distinct substrates such as colloidal chitin and fluorescent 4-Methylumbelliferyl β-D-N,N′,N″-triacetylchitotrioside in our study. The identity of the enriched proteins was corroborated by mass spectrometry as chitinases, evidencing the enrichment of chitinase activity by acetone precipitation. The confirmation of the identity of these chitinases as enriched activities is quite important due to the fast and easy isolation process and their potential use for the biocontrol of phytopathogens that affect crops of commercial interest.

Much of the biotechnological research has focused on the search for chitin-producing microorganisms, mainly due to their ability to control phytopathogenic fungi, which cause damage and economic losses in agriculture [57], as well as to the possibility of replacing pesticides, which are chemically formulated and synthesized and cause negative effects on the environment and human health [58].

In our study, the chitinase produced by *Curtobacterium* sp. CBMAI 2942 was shown to inhibit the growth of *Aspergillus* sp. series *Nigri* CBMAI 1846, isolated from tomato plants, possibly due to the presence of chitin in the composition of the cell wall of the fungus, being able to interfere in morphogenesis, cell division, and reconstitution of the cell wall and directly influencing the nutrition of the phytopathogen [59]. On the other hand, the enzyme did not inhibit the growth of *Botrytis cinerea* CBMAI 0863, *Fusarium complex incarnatum-equisetii* CBMAI 1981, and *Fusarium complex oxysporum* CBMAI 1274. Chitinase produced by *Pseudomonas* GWSMS-1 inhibited the growth of the fungi *Verticillium dahlia* CICC 2534 and *Fusarium oxysporum* f. sp. *cucumerinum* CICC 2532 [10]. Also, the enzyme produced by *Chitinophaga* sp. S167 exhibited inhibitory activity against *Fusarium oxysporum*, *Alternaria alternata,* and *Cladosporium* sp. [59], and the chitinase from *Paenibacillus elgii* PB1 showed antagonistic activity against *Aspergillus niger* (MTCC 282), *Trichophyton rubrum* (MTCC 791), *Microsporum gypseum* (MTCC 2819), and *Candida albicans* (MTCC 227) [60]. The antifungal potential of chitinases obtained from different bacteria is related to the morphology and constitution of chitin in fungal cell walls [61].

In this study, the chitinase production by the psychrotolerant bacterial strain *Curtobacterium* sp. CBMAI 2942, isolated from a bryozoan sample in Maritime Antarctica was investigated using a polyphasic approach. The genetic potential of *Curtobacterium* sp. CBMAI 2942 for chitin degradation was confirmed by genomic analysis, which revealed the presence of two chitin-binding domains (ChBDs) (Chi C). Colloidal chitin, peptone, and KH_2_PO_4_ were the independent variables that showed a statistically significant effect on chitinase production. The enzyme extract showed the enrichment of chitinase activities confirmed by mass spectrometry, and this activity resulted in significant inhibition against *Aspergillus* sp. series *Nigri* CBMAI 1846, a phytopathogenic fungus of tomato plants, suggesting that this bacterium is a good candidate for future use in biological control of agricultural pests.

## 4. Materials and Methods

The flowchart illustrating the experimental design and the analyses carried out in this study is shown in Figure S6.

### 4.1. Bacterial Strain

The bacterial strain under study, *Curtobacterium* sp. CBMAI 2942, originally named *Curtobacterium* sp. 458, was isolated from a bryozoan sample collected on Livingston Island, Maritime Antarctica [62], and it was previously selected as a promising chitinase producer [17]. The bacterial strain was cryopreserved at −80 °C (in 10% glycerol) and kindly provided by the Brazilian Collection of Environmental and Industrial Microorganisms (CBMAI), at the Research Center for Chemistry, Biology and Agriculture (CPQBA), University of Campinas (UNICAMP).

### 4.2. Sequencing, Assembly, and Annotation of the Curtobacterium sp. CBMAI 2942 Genome

#### 4.2.1. Genomic DNA Extraction and Sequencing

Genomic DNA was extracted from a bacterial culture grown on R2A medium for 72 h, the same medium used to isolate the strain [62], employing the PowerMax Soil DNA kit (Mo Bio Laboratories, Carlsbad, CA, USA) and according to the manufacturer’s instructions. The DNA concentration was determined by fluorometry using Qubit (Qubit™ 3.0, Invitrogen), and the purity was estimated by calculating the A260/A280 ratio in a spectrophotometer (NanoDrop™ 1000, Thermo Fisher Scientific 3411 Silverside Road Bancroft Building, Suite 100 Wilmington, DE, USA). Agarose gel electrophoresis (1%) was performed to assess the integrity of the DNA samples. Genomic DNA was processed at the Central Laboratory of High-Throughput Technologies (LaCTAD) of UNICAMP (Campinas, Brazil), and paired and matched DNA libraries were prepared with Nextera XT kit and sequenced on the Illumina Miseq platform.

#### 4.2.2. Assembly and Annotation

The quality of the raw reads was assessed using the FastQC 0.11.9 tool, and the output allowed us to identify and remove adapters and primers, low-quality endpoints, and to filter reads with an average quality (Phred) less than 30 by running Trimmomatic v0.39 software [63,64]. In addition, reads with lengths smaller than 100 bp were discarded. All sequences that passed quality control were used for the genome assembly. For this, Spades v3.13 [65] was used with both types of reads and with different values of k-mers (21 to 127). The quality of the assembly, based on the N50 and the number of contigs, was evaluated using the Quast v5.0.2 package [66]. Genome completeness and contamination were assessed with the CheckM v1.1.3 tool. All contigs smaller than 600 bp in length were removed for the next steps.

Gene prediction was performed using Prodigal v2.6.3 [67]. The detected genes were annotated with KEGG (Kyoto Encyclopedia of Genes and Genomes) [68] and eggNOG (database of orthology relationships, functional annotation, and evolutionary histories of genes) [69] databases using the Diamond sequence aligner v0.9.14 [70]. In addition, files containing the predicted genes were submitted to the eggNOG-mapper v2 web-based tool [71].

#### 4.2.3. Phylogenomic Identification

To reconstruct the evolutionary relationships of the sequenced bacterial strain, a phylogenomic approach was employed following the PhyloPhlAn v3.0 pipeline [72]. This tool allows us to perform complete phylogenetic analyses with genomic data. Genomes of the most closely reference strains related to the bacterium of interest were retrieved from the NCBI (National Center for Biotechnology Information) RefSeq database. The pipeline selects a relevant set of phylogenetic markers (400) to search across genome sequences using Diamond v0.9.21 [70]. The sequences of the detected genes were aligned with the MAFFT v7.487 multiple sequence alignment tool [73]. Marker alignments were concatenated to perform the phylogeny reconstruction using FastTree v2.1.11 [74]. The resulting tree was visualized and customized in the iTOL web tool (http://itol.embl.de accessed 4 March 2022) [75]. OGRIs (Overall Genome-Related Indices) were calculated pairwise with the assembled genome of *Curtobacterium* sp. CBMAI 2942 and the closest reference genomes to define the species boundaries. Specifically, ANI (Average Nucleotide Identity) and dDDH (digital DNA–DNA hybridization) were calculated by submitting genome sequences in the web-based tools JSPeciesWS (https://jspecies.ribohost.com/jspeciesws/#analyse and accessed 4 March 2022) and Genome-to-Genome Distance Calculator 3.0 (https://ggdc.dsmz.de/ggdc.php# accessed 4 March 2022), respectively. Values greater than 95% of ANI and 70% of dDDH indicate that the individuals belong to the same species.

### 4.3. Production of Chitinase in Liquid Medium

#### 4.3.1. Cultivation Conditions

A standardized inoculum of 1 × 10^6^ cells/mL of the chitinase-producing bacterium was prepared with saline solution (NaCl 0.9%). A volume of 30 mL of this inoculum was added to 270 mL of culture medium (in g/L: peptone 0.3, yeast extract 0.3, K_2_HPO_4_ 0.7, KH_2_PO_4_ 0.3, MgSO_4_·7H_2_O 0.5, and colloidal chitin 1%) and placed in a 1 L Erlenmeyer flask. Colloidal chitin was obtained as described by Vazques et al. [17], with a final pH of 7.0 to 7.2. The growth culture was incubated at 15 °C and 150 rpm for 72 h.

#### 4.3.2. Determination of Biomass

Biomass was evaluated by optical density (OD), read in a spectrophotometer at 600 nm, and counts of colony forming units (CFUs) after serial dilution (10^−1^ to 10^−6^) of bacterial growth and plating on culture medium with colloidal chitin. CFU results were used as input data in the OriginPro Ver.8 software with the modified Gompertz mathematical model for bacterial growth analysis, generating the growth rate [(CFU/mL)/time], adaptation time (lag phase), and generation time (log phase).

#### 4.3.3. Quantification of Total Proteins

Total proteins were measured using 2,2′-Biquinoline-4,4′-dicarboxylic acid (or 2,2′-Bicinchoninic acid, BCA) with the kit BCA Protein Assay (Thermo Scientific^®^, Product No. 23225, Rockford, IL, USA). Assays were performed in a 96-well deep well plate containing 25 μL of the cell-free centrifuged supernatant (enzymatic broth) and 200 μL of BCA reagent, and the reaction was incubated at 37 °C for 30 min and read by absorbance at 562 nm. For the standard protein curve (Figure S7), bovine serum albumin (BSA) was used, and analyses were carried out in triplicate.

#### 4.3.4. Chitinase Enzyme Activity

The enzymatic activity was evaluated based on the method modified by Ulhoa and Peberdy [76], consisting of the quantification of N-Acetylglucosamine (GlcNAc), using 3,5-Dinitrosalicylic acid (DNS), for which a standard curve was used (Figure S8) with N-Acetylglucosamine (GlcNAc), R^2^ = 0.9881. One unit of enzyme activity (U) was expressed in µmol of N-Acetylglucosamine/mL/hour using the formula:

$$Chitinase\;enzyme\;activity\left(U\right)=\frac{CGlc.Vt}{PM.Ve.T(h)}.Dil$$

CGlc = concentration of N-acetylglucosamine (GlcNAc) determined by the standard curve.
Vt = total volume in the enzymatic reaction (2 mL).
PM = N-acetylglucosamine (GlcNAc) molecular mass (221.208 mg/mmol).
Ve = volume of enzyme preparation used (1 mL).
T = reaction time (6 h).
Dil = dilution applied to the enzyme preparation.

Alternatively, enzyme activity was measured using 4-Methylumbelliferyl β-D-N,N′,N″-triacetylchitotrioside (Merck^®^) 140 µM in the soluble fraction and citrate–phosphate 0.1 M buffer pH 5 °C at 30 °C. The reaction was interrupted by the addition of Gly-NH_4_OH 0.8 M buffer pH 10. Methylumbelliferone fluorescence was measured in a Gemini XPS with an excitation wavelength of 320 nm and an emission wavelength of 420 nm.

#### 4.3.5. Evaluation of the Optimal Growth Temperature

The growth conditions were carried from 5 °C to 40 °C (5 °C spacing) to determine the optimal growth temperature for 72 h, and biomass, total protein quantification, and chitinase activity were evaluated.

### 4.4. Statistical Optimization Design for Chitinase Production

The strategy used was composed of two Plackett–Burman designs (PB16 and PB12) for variable selection and range, and one Central Composite Design (CCD2^3^). In the end, the assay with the highest enzymatic activity was validated. The program used for experimental design was Protimiza Experimental Design software (http://experimental-design.protimiza.com.br accessed on 23 May 2024).

#### 4.4.1. Screening of Variables by Plackett–Burman Design

The Plackett–Burman design, PB16 (16 experiments plus 3 central points), was initially carried out to analyze the effect of nine variables (colloidal chitin, peptone, yeast extract, K_2_HPO_4_, KH_2_PO_4_, MgSO_4_·7H_2_O, NH_4_NO_3,_ NaCl, and pH) (Table S9). After discarding the variables with a negative effect (*p* > 0.1), the second Plackett–Burman, a PB12 (12 experiments with 3 central points), was performed to evaluate the effect of four variables (chitin, peptone, KH_2_PO_4_, and MgSO_4_·7H_2_O) with a significance level of *p* < 0.05 (Table 3). These assays were performed at 25 °C and 150 rpm for 96 h and 120 h, and the standardized effect was based on the following first-order model: y = β0 + Σβixi, where y is the predicted response, β0 is the model intercept, βi is the linear coefficient, and xi is the independent variable level. Chitinase production was the response variable evaluated in all experimental designs.

#### 4.4.2. Central Composite Design

After analysis of the Plackett–Burman (PB) design, a Central Composite Design (CCD 2^3^) was performed for three independent variables (colloidal chitin, peptone, and KH_2_PO_4_), with a total of 14 experiments and 3 central points, totaling 17 experiments to improve chitinase production by the selected strain. These experiments were performed at pH 6.0, 25 °C, and 150 rpm. The response variable was chitinase production at two incubation times (96 h and 120 h), using *p* < 0.05. The data were analyzed using analysis of variance (ANOVA) followed by Fisher’s test, and the model equation was confirmed by the determination of the R^2^ coefficient at 120 h.

The response surface plot was generated to analyze the effect of independent variables on the chitinase production in CCD 2^3^ by *Curtobacterium* sp. CBMAI 2942. The validation assay was with colloidal chitin (6.98%), peptone (0.40 g/L), KH_2_PO_4_ (0.35 g/L), and MgSO_4_ (0.5 g/L), and the response variables were chitinase production, as well as total proteins and final pH of the fermentation process during up to 144 h of incubation at 25 °C and 150 rpm.

### 4.5. Separation by SDS-PAGE and Identification of Chitinases via Mass Spectrometry

Cell cultures were centrifuged at 10,000 rpm, and supernatant was used as an enzyme source. Enriched chitinase samples were 1:1 mixed into sample buffer (60 mM Tris–HCl buffer (pH 6.8), 2.5% SDS, 0.36 mM b-mercaptoethanol, 10% (*v/v*) glycerol, and 0.005% (*w/v*) bromophenol blue; thereafter, the sample was heated at 100 °C for 5 min, and 20 µL was applied on a 12% polyacrilamide gel (40%T and 2.7%C) using BioRad Mini-Protean II System (2000 Alfred Nobel Drive Hercules, CA, USA). Sample electrophoretic separation was performed at a constant voltage of 200 V, and the gel was stained with silver and sliced for analysis. Proteomic analysis was performed with a gel slice of approximately 1 mm^2^, and the pieces were rinsed out with 200 µL of destaining solution (50 mM of NH_4_HCO_3_ in 25% acetonitrile (ACN)). In the next step, 200 µL of ACN was added until the gel was completely destained, and in sequence, ACN was removed by vacuum centrifugation (Speed Vac) for 10 min. After that, 100 mM of dithiothreitol (DTT) was added (*v*/*v*) and incubated for 30 min at room temperature. After incubation, the DTT solution was discarded. The addition of ACN and vacuum centrifugation was repeated. The sample was then alkylated with 200 mM (*v*/*v*) of iodoacetamide (IAA) at 25 °C for 30 min in the dark. After this procedure, the IAA solution was discarded, and the addition of ACN was repeated once more. Finally, samples were incubated for 18 h with 100 ng of bovine pancreatic trypsin (Sigma 6502), and the reaction was stopped with 10 µL of 10% acetic acid and concentrated by vacuum centrifugation. The samples were suspended in 50 μL of formic acid (FA 0.1%) for a further mass spectrometry procedure.

Tandem mass spectrometry analysis of tryptic peptides was performed using an LC–MS/MS IT-TOF (Shimadzu). Samples (50 µL aliquot) were loaded into a C18 column (Gemini C18, 110A; 50 × 2.0 mm), and peptides were eluted by a binary gradient of 5% to 40%, solvent A—water/FA (999:1) and solvent B—ACN/water/FA (900:99:1), at a constant flow of 0.2 mL/min for 40 min. The raw data were converted to an mzXML file and loaded into Peaks Studio V7.0 (BSI, Canada). The data were processed according to the following parameters: MS and MS/MS error mass were 0.1 Da; methionine oxidation and carbamidomethylation as variable and fixed modification, respectively; trypsin as cleaving enzyme; maximum missed cleavages (3), maximum variable PTMs per peptide (3), and non-specific cleavage (both); ion source: ESI (nano-spray); and fragmentation mode CID, CAD (y and b ions).

### 4.6. Antifungal Activity of Chitinases

The evaluation of the inhibition of mycelial growth by the chitinase produced by *Curtobacterium* sp. CBMAI 2942 was carried out using paper discs, according to Liu et al. [10]. Initially, sterilized discs of filter paper of 6.0 mm diameter were immersed in the enzyme extract for 5 min and then placed in the center of Petri dishes containing sweet potato dextrose agar (potato dextrose agar—PDA). The mycelium of phytopathogenic fungi with 6.0 mm in diameter was placed around the filter paper immersed in the enzyme extract, and the plates were incubated at 28 °C for 7 days. Negative control with acetone and positive control with the commercial fungicide Itraconazole (10 mg/L) were used. The phytopathogenic fungi used were *Fusarium incarnatum* CBMAI 1981 (mango pathogen), *Fusarium complex fujikuroi* CBMAI 1274 (soil isolate), *Botrytis cinerea* CBMAI 0863 (grape isolate), and *Aspergillus* sp. series *Nigri* CBMAI 1846 (tomato isolate). All fungal strains were kindly provided by CBMAI.

## Figures and Tables

**Table 1:** Genome assembly statistics of marine *Curtobacterium* sp. CBMAI 2942.

Sequence Data	*Curtobacterium* sp. CBMAI 2942
**Coverage (X)**	350
**# Contigs**	1
**Total compression**	3,685,083
**Largest *contig***	3,679,616
**Smallest *contig***	5467
**N50**	3,679,616
**% CG**	71.48
**% Completeness**	99.44
**% Contamination**	0.76

**Table 2:** Calculations of ANI and dDDH values between *Curtobacterium* sp. CBMAI 2942 and related bacteria based on the complete genome.

Marine Bacterial Strain	Related Bacteria	ANIb (%)	dDDH (%)	Difference in G + C Content (%)
*Curtobacterium* sp. CBMAI 2942	*Curtobacterium luteum* DSM 20542^T^	87.71	33.60	0.22
	*Curtobacterium oceanosedimentum* NS359	82.66	26.10	0.26
	*Curtobacterium citreum* DSM 20528^T^	82.79	26.60	0.46
	*Curtobacterium pusillum* ATCC 19096^T^	83.52	27.00	0.63

**Table 3:** Data of the two domains of chitin-binding genes present in *Curtobacterium* sp. CBMAI 2942.

Bacterium	Gene ID	Gene Name	Start	Final	Size (pb)	COG ID	Function COG
*Curtobacterium* sp. CBMAI 2942	>NODE_1_length_3679616_cov_83.786059_76	Chitin binding	91,936	93,246	1310	COG3979	ChBD of chitin binding (Chi C)
	>NODE_1_length_3679616_cov_83.786059_3152	Chitin binding	3,317,000	3,318,553	1553	COG3979	ChBD of chitin binding (Chi C)

**Table 4:** Plackett–Burman design matrix (PB) with 16 trials and three central points and used to evaluate the effect of nine variables on chitinase production by *Curtobacterium* sp. CBMAI 2942 (after 96 h and 120 h at 25.0 °C and 150 rpm).

Assay	Colloidal Chitin (%)	Yeast Extract (g/L)	Peptone (g/L)	K_2_HPO_4_ (g/L)	KH_2_PO_4_ (g/L)	MgSO_4_·7H_2_O (g/L)	NH_4_NO_3_ (g/L)	NaCl (g/L)	pH	U/L (96 h)	U/L (120 h)
1	1 (3)	−1 (0.1)	−1 (0.1)	−1(0.2)	1 (0.5)	−1 (0.1)	−1 (0.2)	1 (1.8)	1(8)	28.18	28.96
2	1 (3)	1 (0.5)	−1 (0.1)	−1(0.2)	−1 (0.1)	1 (0.9)	−1 (0.2)	−1 (0.2)	1(8)	31.89	38.54
3	1 (3)	1 (0.5)	1 (0.5)	−1(0.2)	−1 (0.1)	−1 (0.1)	1 (3.8)	−1 (0.2)	−1 (6)	30.72	32.67
4	1 (3)	1 (0.5)	1 (0.5)	1 (1.2)	−1 (0.1)	−1 (0.1)	−1 (0.2)	1 (1.8)	−1 (6)	28.18	29.15
5	−1 (1)	1 (0.5)	1 (0.5)	1 (1.2)	1 (0.5)	−1 (0.1)	−1 (0.2)	−1 (0.2)	1(8)	28.96	30.52
6	1 (3)	−1 (0.1)	1 (0.5)	1 (1.2	1 (0.5)	1 (0.9)	−1 (0.2)	−1 (0.2)	−1 (6)	39.33	39.72
7	−1 (1)	1 (0.5)	−1 (0.1)	1 (1.2)	1 (0.5)	1 (0.9)	1 (3.8)	−1 (0.2)	−1 (6)	30.52	32.09
8	1 (3)	−1 (0.1)	1 (0.5)	−1(0.2)	1 (0.5)	1 (0.9)	1 (3.8)	1 (1.8)	−1 (6)	44.22	46.76
9	1 (3)	1 (0.5)	−1 (0.1)	1 (1.2)	−1 (0.1)	1 (0.9)	1 (3.8)	1 (1.8)	1(8)	27.98	28.18
10	−1 (1)	1 (0.5)	1 (0.5)	−1(0.2)	1 (0.5)	−1 (0.1)	1 (3.8)	1 (1.8)	1(8)	30.72	32.09
11	−1 (1)	−1 (0.1)	1 (0.5)	1 (1.2)	−1 (0.1)	1 (0.9)	−1 (0.2)	1 (1.8)	1(8)	30.52	32.28
12	1 (3)	−1 (0.1)	−1 (0.1)	1 (1.2)	1 (0.5)	−1 (0.1)	1 (3.8)	−1 (0.2)	1(8)	28.18	30.33
13	−1 (1)	1 (0.5)	−1 (0.1)	−1(0.2)	1 (0.5)	1 (0.9)	−1 (0.2)	1 (1.8)	−1 (6)	25.83	26.22
14	−1 (1)	−1 (0.1)	1 (0.5)	−1(0.2)	−1 (0.1)	1 (0.9)	1 (3.8)	−1 (0.2)	1(8)	26.81	32.09
15	−1 (1)	−1 (0.1)	−1 (0.1)	1 (1.2)	−1 (0.1)	−1 (0.1)	1 (3.8)	1 (1.8)	−1 (6)	25.24	26.22
16	−1 (1)	−1 (0.1)	−1 (0.1)	−1(0.2)	−1 (0.1)	−1 (0.1)	−1 (0.2)	−1 (0.2)	−1 (6)	26.02	28.57
17	0 (2)	0 (0.3)	0 (0.3)	0 (0.7)	0 (0.3)	0 (0.5)	0 (2.0)	0 (1.0)	0 (7)	27.20	27.98
18	0 (2)	0 (0.3)	0 (0.3)	0 (0.7)	0 (0.3)	0 (0.5)	0 (2.0)	0 (1.0)	0 (7)	27.78	28.18
19	0 (2)	0 (0.3)	0 (0.3)	0 (0.7)	0 (0.3)	0 (0.5)	0 (2.0)	0 (1.0)	0 (7)	27.00	28.96

**Table 5:** Central Composite Design (CCD 2^3^) to production of chitinase by *Curtobacterium* sp. CBMAI 2942 at 96 h and 120 h of incubation at 25.0 °C and 150 rpm.

Assay	Colloidal Chitin (%)	Peptone (g/L)	KH_2_PO_4_ (g/L)	U/L (96 h)	U/L (120 h)
1	(1.50)	(0.20)	(0.20)	32.09	35.41
2	(5.00)	(0.20)	(0.20)	58.50	71.80
3	(1.50)	(0.60)	(0.20)	30.33	32.28
4	(5.00)	(0.60)	(0.20)	60.26	80.01
5	(1.50)	(0.20)	(0.50)	31.70	34.83
6	(5.00)	(0.20)	(0.50)	54.00	71.21
7	(1.50)	(0.60)	(0.50)	30.13	31.50
8	(5.00)	(0.60)	(0.50)	61.82	75.91
9	(0.31)	(0.40)	(0.35)	23.28	24.46
10	(6.19)	(0.40)	(0.35)	86.27	99.19
11	(3.25)	(0.06)	(0.35)	36.78	42.26
12	(3.25)	(0.74)	(0.35)	38.74	39.72
13	(3.25)	(0.40)	(0.10)	39.52	41.09
14	(3.25)	(0.40)	(0.60)	58.69	59.28
15	(3.25)	(0.40)	(0.35)	57.32	58.69
16	(3.25)	(0.40)	(0.35)	57.13	61.04
17	(3.25)	(0.40)	(0.35)	56.54	58.89

## Data Availability

The genome sequences were deposited in the GenBank database under the accession numbers CP129104 and BioProject PRJNA977061.

## Supplementary Materials

The following supporting information can be downloaded at: https://www.mdpi.com/article/10.3390/ijms25179250/s1.
