# Supplementary material for: Genomics, Proteomics, and Antifungal Activity of Chitinase from the Antarctic Marine Bacterium Curtobacterium sp. CBMAI 2942
Source: Int J Mol Sci. 2024 Aug 26;25(17):9250. doi: 10.3390/ijms25179250 (PMC11395076; doi:10.3390/ijms25179250)
Supplement: Supplementary file 1 [file ijms-25-09250-s001.zip › ijms-3119275-supplementary.pdf]

International Journal of
Molecular Sciences

MDPI

Supplementary Material

Genomics, proteomics and, antifungal activity of chitinase from the Antarctic marine bacterium *Curtobacterium* sp. CBMAI 2942

Yesenia Melissa Santa-Cruz Vasquez[1,2], Luis Gabriel Cueva-Yesquen [1,2], Alysson Wagner Fernandes Duarte[3*], Luiz Henrique Rosa[4], Rodrigo Valladão[5], Adriana Rios Lopes[5], Rafaella Costa Bonugli-Santos[6], Valéria Maia de Oliveira[1*]

Figure S1. Diagram of the position of the chitin binding domain in the genome of *Curtobacterium* sp. CBMAI 2942.

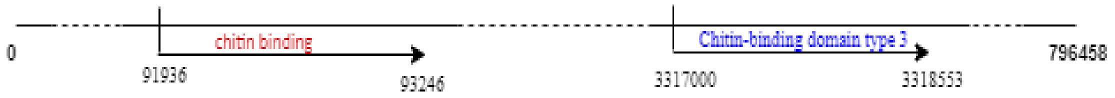

Figure S2. Plot of experimental vs. predicted values for chitinase production by *Curtobacterium* sp. CBMAI 2942 for CCD 2^3 design. A) 96h; B) 120h.

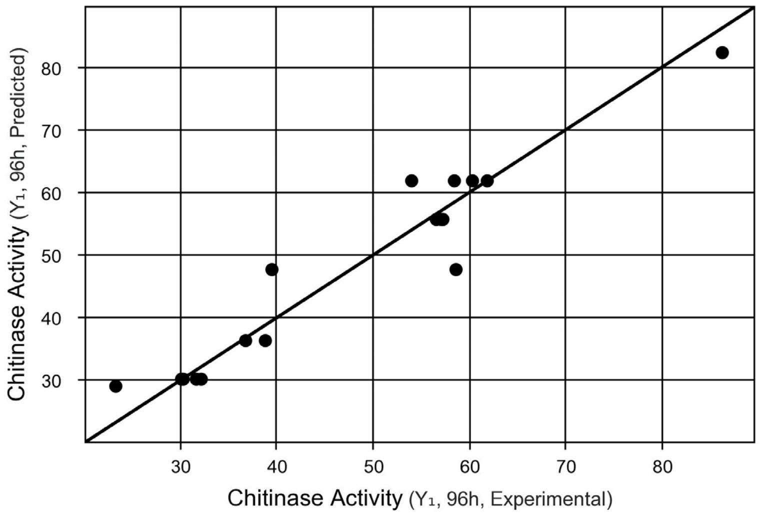

A)

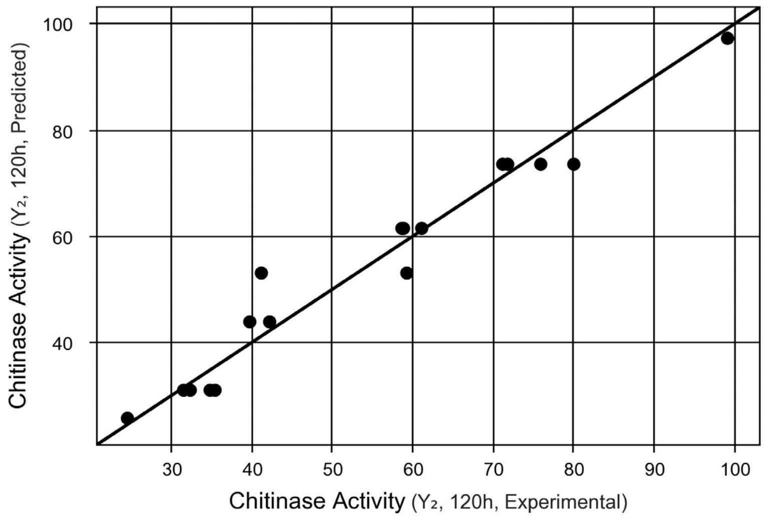

B)

Figure S3. Desirability functions for chitinase production by *Curtobacterium* sp. CBMAI 2942 after 120 hours of incubation based on the CCD 2^3 experiment design. Each response is converted into an individual desirability function, ranging from 0 (completely undesirable) to 1 (optimal). Independent variables are selected to maximize overall desirability. The graphs in the last row display the global desirability profiles, with vertical lines indicating the conditions that achieve maximum global desirability.

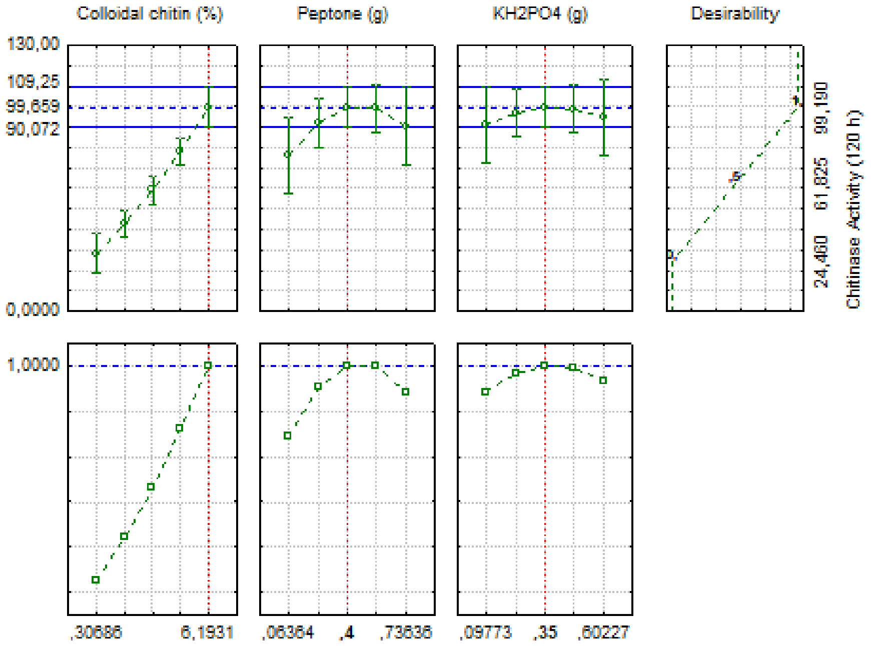

Figure S4. Antifungal activity test with enzyme extract precipitated with acetone (1:5) against A) *Aspergillus* sp. series *nigri* CBMAI 1846, B) *Botrytis cinerea* CBMAI 0863 c) *Fusarium complex incarnatum-equisetii* CBMAI 1981 D) *Fusarium complex oxysporum* CBMAI 1274, after 7 days of incubation.

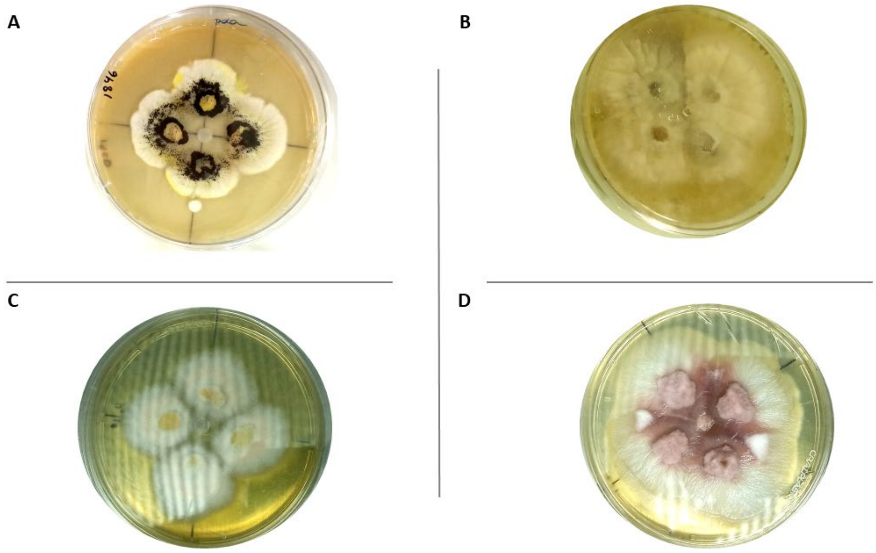

Figure S5. Microscopic analysis of the mycelium of *Aspergillus* sp. series *nigri* CBMAI 1846 after the antifungal test with the enzyme crude extract precipitated with acetate (dilution 1:5). (A) Control (mycelium treated with acetone and water); (B) Treatment with enzyme extract of *Curtobacterium* sp. 458, showing the damaged hyphae.

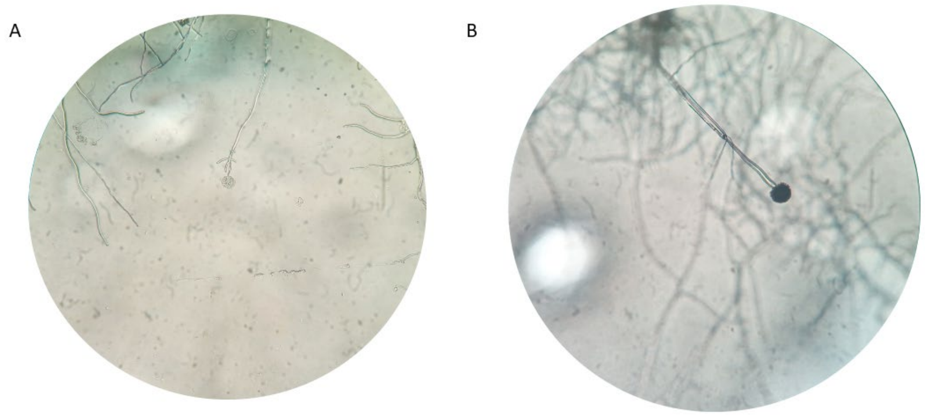

Figure S6. Flowchart of the steps of the present study.

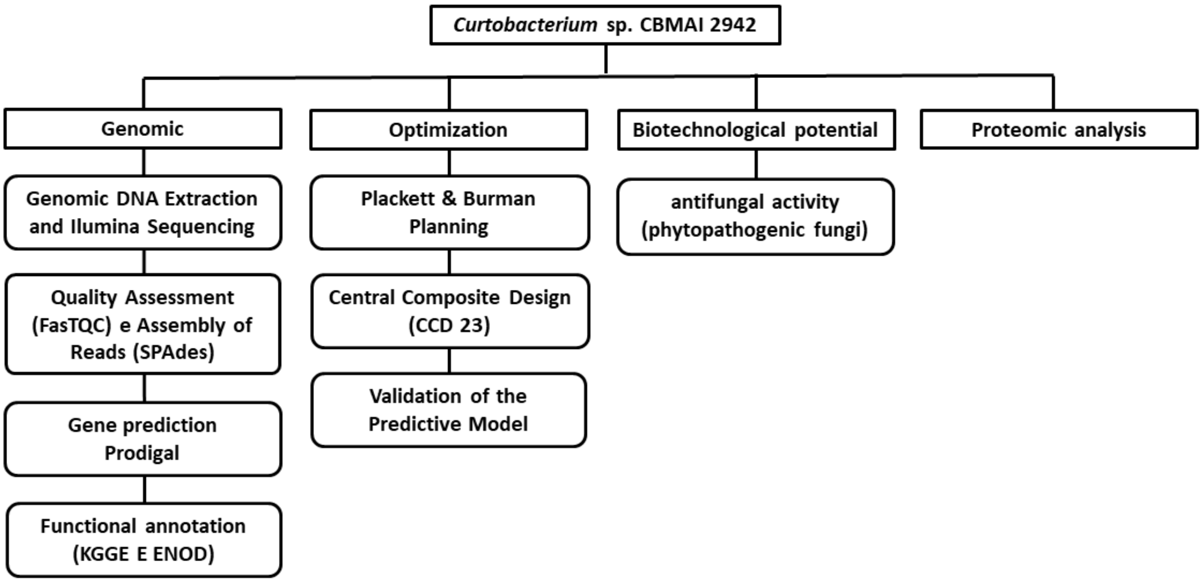

Figure S7. Bovine serum albumin (BSA) standard curve by the BCA kit used for total protein quantification.

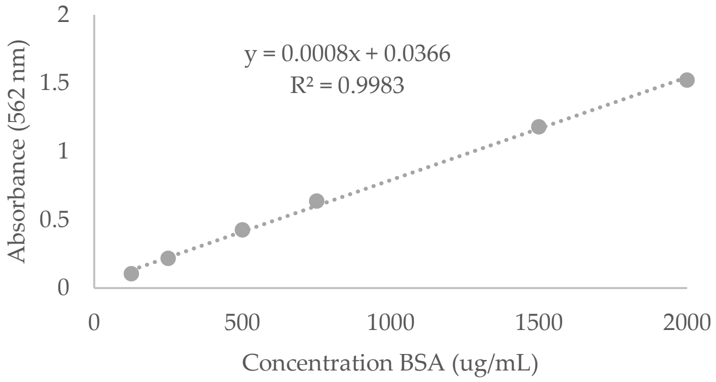

Figure S8. Standard curve for determination of reducing sugars by DNS using N-Acetylglucosamine (GlcNAc).

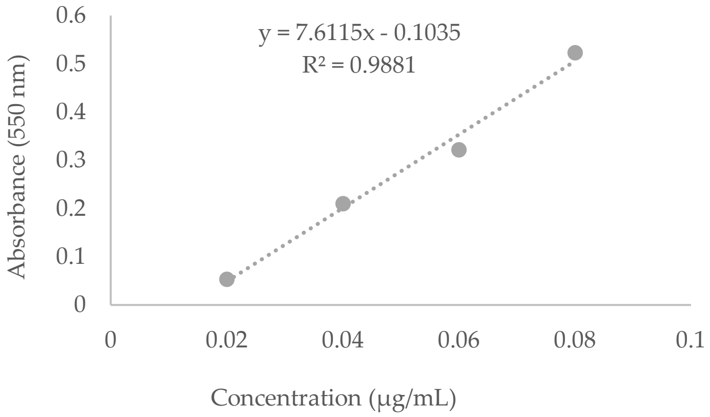

Table S1. Standardized effects for Placket-Burman (P&B16) for chitinase activity produced by *Curtobacterium* sp. CBMAI 2942 at 25°C and 150 rpm ($p < 0.1$) by 96 hours incubation.

Name	Effect	Standard Error	Calculated	p-value
**Mean**	32.15	0.96	33.59	0.0000
**Curvature**	-7.55	4.82	-1.57	0.1556
**Colloidal chitin (x_1)**	4.28	1.91	2.24	0.0558*
**Yeast extract (x_2)**	-1.93	1.91	-1.01	0.3420
**Peptone (x_3)**	4.52	1.91	2.36	0.0458*
**K_2HPO_4 (x_4)**	-2.18	1.91	-1.14	0.2885
**KH_2PO_4 (x_5)**	2.37	1.91	1.24	0.2501
**$MgSO_4.7H_2O$ (x_6)**	4.67	1.91	2.44	0.0406*
**NH_4NO_3 (x_7)**	0.81	1.91	0.42	0.6838
**NaCl (x_8)**	-1.83	1.91	-0.96	0.3662
**pH (x_9)**	-1.05	1.91	-0.55	0.5979

Table S2. Standardized effects for Placket-Burman (P&B16) for chitinase activity produced by *Curtobacterium* sp. CBMAI 2942 at 25°C and 150 rpm ($p < 0.1$) by 120 hours incubation.

Name	Effect	Standard Error	Calculated	p-value
**Mean**	30.21	0.79	38.36	0.0000
**Curvature**	-5.76	3.96	-1.45	0.1843
**Colloidal chitin (x_1)**	4.26	1.57	2.70	0.0269*
**Yeast extract (x_2)**	-1.71	1.57	-1.09	0.3085
**Peptone (x_3)**	4.45	1.57	2.83	0.0222*
**K2HPO4 (x_4)**	-0.68	1.57	-0.43	0.6751
**KH2PO4 (x_5)**	3.57	1.57	2.27	0.0530*
**MgSO4.7H2O (x_6)**	3.86	1.57	2.45	0.0398*
**NH4NO3 (x_7)**	0.68	1.57	0.43	0.6751
**NaCl (x_8)**	-0.19	1.57	-0.12	0.9045
**pH (x_9)**	-2.10	1.57	-1.34	0.2186

Table S3. Plackett & Burman design (P&BP12) used to evaluate the effect of four variables on chitinase and total protein production by *Curtobacterium* sp. CBMAI 2942 at 96 h and 120 h of incubation at 25°C and 150 rpm.

Assay	Colloidal chitin (%)	Peptone (g/L)	KH_2PO_4 (g/L)	$MgSO_4.7H_2O$ (g/L)	U/L (96 h)	U/L (120 h)
**1**	1 (4)	-1 (0.3)	1 (0.7)	-1 (0.5)	59.08	59.47
**2**	1 (4)	1 (0.7)	-1 (0.3)	1 (1.5)	57.52	59.28
**3**	-1 (2)	1 (0.7)	1 (0.7)	-1 (0.5)	30.91	35.22
**4**	1 (4)	-1 (0.3)	1 (0.7)	1 (1.5)	61.43	67.69
**5**	1 (4)	1 (0.7)	-1 (0.3)	1 (1.5)	60.65	60.65
**6**	1 (4)	1 (0.7)	1 (0.7)	-1 (0.5)	63.78	70.43
**7**	-1 (2)	1 (0.7)	1 (0.7)	1 (1.5)	28.18	34.43
**8**	-1 (2)	-1 (0.3)	1 (0.7)	1 (1.5)	38.74	46.76
**9**	-1 (2)	-1 (0.3)	-1 (0.3)	1 (1.5)	38.15	45.00
**10**	1 (4	-1 (0.3)	-1 (0.3)	-1 (0.5)	66.52	66.54
**11**	-1 (2)	1 (0.7)	-1 (0.3)	-1 (0.5)	33.26	37.76
**12**	-1 (2)	-1 (0.3)	-1 (0.3)	-1 (0.5)	38.35	41.28
**13**	0 (3)	0 (0.5)	0 (0.5)	0 (1.0)	44.41	48.91
**14**	0 (3)	0 (0.5)	0 (0.5)	0 (1.0)	42.26	45.39
**15**	0 (3)	0 (0.5)	0 (0.5)	0 (1.0)	45.19	51.65

Table S4. Standardized effects for Placket-Burman (P&B12) for chitinase produced by *Curtobacterium* sp. CBMAI 2942 at 25°C and 150 rpm ($p < 0.05$) by 96 hours incubation.

Name	Effect	Standard Error	Calculated	p-value (<0.05)
**Mean**	48.05	0.84	57.41	0.0000
**Curvature**	-8.19	3.74	-2.19	0.0565
**Colloidal Chitin (x_1)**	26.90	1.67	16.07	0.0000*
**Peptone (x_2)**	-4.66	1.67	-2.79	0.0212*
**KH2PO4 (x_3)**	-2.05	1.67	-1.23	0.2507
**MgSO4.7H2O (x_4)**	-1.21	1.67	-0.72	0.4898

Table S5. Standardized effects for Placket-Burman (P&B12) for chitinase produced by *Curtobacterium* sp. CBMAI 2942 at 25°C and 150 rpm ($p < 0.05$) by 120 hours incubation.

Name	Effect	Standard Error	Calculated	p-value ($p < 0.1$)
**Mean**	52.04	1.34	38.77	0.0000
**Curvature**	-6.78	6.00	-1.13	0.2875
**Collodial Chitin (x_1)**	23.94	2.68	8.92	0.0000*
**Peptone (x_2)**	-4.83	2.68	-1.80	0.1056
**KH2PO4 (x_3)**	0.58	2.68	0.22	0.8333
**MgSO4.7H2O (x_4)**	0.52	2.68	0.19	0.8512

Table S6. Regression coefficient for CCD 2^3 for chitinase production by *Curtobacterium* sp. CBMAI 2942 at 25°C and 150 rpm by 96 hours incubation.

Factor	Regression coefficient	Error Standard	T value	P value ($p < 0.05$)
**Mean**	52.81	1.82	28.99	0.0000
**Colloidal chitin (L)**	15.84	1.50	10.58	0.0000
**Peptone (Q)**	-6.20	1.53	-4.04	0.0012

L = Linear, Q = Quadratic

Table S7. Regression coefficient for CCD 2^3 for chitinase production by *Curtobacterium* sp. CBMAI 2942 at 25°C and 150 rpm ($p < 0.05$) by 120 hours incubation.

Factor	Regression coefficient	Error Standard	T value	P value ($p < 0.05$)
**Mean**	58.43	1.81	32.30	0.0000
**Colloidal chitin (L)**	21.28	1.49	14.31	0.0000
**Peptone (Q)**	-5.55	1.52	-3.64	0.0027

L = Linear, Q = Quadratic

Table S8. Quadratic model analysis for chitinase production by *Curtobacterium* sp. CBMAI 2942 at 25°C and 150 rpm ($p < 0.05$) by 120 hours incubation. R^2=95.53%

Variation Source	Sum of squares	Degrees of freedom	Mean square	F calc	p-valor
**Regression**	6584.4	2	3292,2	109	0.0000
**Residuals**	422.8	14	30,2		
**Lack of Fit**	419.4	12	34,9	20.6	0.04722
**Pure Error**	3.4	2	1,7		
**Total**	7007.2	16			

Coefficient of determination (R^2)=94%

F 2, 14, 0.05 (F tabulated)= 2.56

Table S9. Codified and real values of the 9 independent variables used in the Placket-Burman design for chitinase production.

Variables	Lower level (-1)	Central point (0)	Higher level (+1)
**Colloidal chitin (%)**	1.0	2.0	3.0
**Peptone (g/L)**	0.1	0.3	0.5
**Yeast extract (g/L)**	0.1	0.3	0.5
**K_2HPO_4 (g/L)**	0.2	0.7	1.2
**KH_2PO_4 (g/L)**	0.1	0.3	0.5
**$MgSO_4.7H_2O$ (g/L)**	0.1	0.5	0.9
**NH_4NO_3 (g/L)**	0.2	2.0	3.8
**NaCl (g/L)**	0.2	1.0	1.8
**pH**	6.0	7.0	8.0
